# Workplace Healthy Lifestyle Determinants and Wellbeing Needs across the Preconception and Pregnancy Periods: A Qualitative Study Informed by the COM-B Model

**DOI:** 10.3390/ijerph18084154

**Published:** 2021-04-14

**Authors:** Seonad K. Madden, Claire A. Blewitt, Kiran D. K. Ahuja, Helen Skouteris, Cate M. Bailey, Andrew P. Hills, Briony Hill

**Affiliations:** 1 School of Health Sciences, College of Health and Medicine, University of Tasmania, Locked Bag 1322, Launceston, TAS 7250, Australia; seonad.madden@utas.edu.au (S.K.M.); andrew.hills@utas.edu.au (A.P.H.); 2 Health and Social Care Unit, School of Public Health and Preventive Medicine, Monash University, 553 St Kilda Road, Melbourne, VIC 3004, Australia; claire.blewitt@monash.edu (C.A.B.); helen.skouteris@monash.edu (H.S.); cate.bailey@monash.edu (C.M.B.)

**Keywords:** women’s health, COM-B, healthy lifestyle behaviors, occupational health

## Abstract

Overweight and obesity present health risks for mothers and their children. Reaching women during the key life stages of preconception and pregnancy in community settings, such as workplaces, is an ideal opportunity to enable health behavior change. We conducted five focus groups with 25 women aged between 25 and 62 years in order to investigate the determinants of healthy lifestyle behaviors, weight management, and wellbeing needs during the preconception and pregnancy periods in an Australian university workplace. Discussions explored women’s health and wellbeing needs with specific reference to workplace impact. An abductive analytical approach incorporated the capability, opportunity, and motivation of behavior (COM-B) model, and four themes were identified: hierarchy of needs and values, social interactions, a support scaffold, and control. Findings highlight the requirement for greater organization-level support, including top-down coordination of wellbeing opportunities and facilitation of education and support for preconception healthy lifestyle behaviors in the workplace. Interventionists and organizational policy makers could incorporate these higher-level changes into workplace processes and intervention development, which may increase intervention capacity for success.

## 1. Introduction

Approximately 50% of women from high-income countries enter pregnancy with overweight or obesity [1] and women of reproductive age typically have low adherence to dietary and physical activity guidelines [2,3]. These traits, while symptomatic of our modern physical and social environment, culminate in heightened chronic disease risk, as well as poorer health outcomes for mothers and their children [4]. Accordingly, the preconception period has been identified as a ‘window of opportunity’ to maximize lifespan health and alleviate weight gain [4]; however, scope for prevention is diminished by women’s lack of engagement with clinical settings pre-pregnancy. Understandably, this is because many women do not view ‘preconception’ as a distinct life stage necessitating change [5,6]. Conversely, for preconception women who do enlist the help of primary care providers, health information and support can be inadequate [7]. Given the high proportion of women of reproductive age in the workforce and the propensity for work to influence our health, workplaces can extol the benefits of health advancement and bridge the gap between relevant health care messaging and preconception healthy lifestyle behaviors (HLB) [8]. Utilization of workplace opportunities to leverage preconception and pregnancy health can also direct attention to the non-medicalized aspects of health and wellbeing, including life satisfaction or personal fulfillment [9], and address the complex interplay between individual motivations, interpersonal workplace relationships, and the physical working environment.

The workplace presents specific challenges to the health and wellbeing of employees, manifested in the form of physical (e.g., heavy workloads) and social opportunities (e.g., workplace culture). We know work factors can contribute to ill health, poor lifestyle behaviors, and psychosocial risks [10,11]. However, the mechanism by which individual factors, such as capacity and motivation, interact with workplace processes to direct the health and wellbeing-related behaviors of preconception and pregnant working women, has yet to be established. This knowledge is needed to inform contextualized workplace behavior change interventions, structured according to women’s evolving needs across the reproductive years.

**Figure 1:**
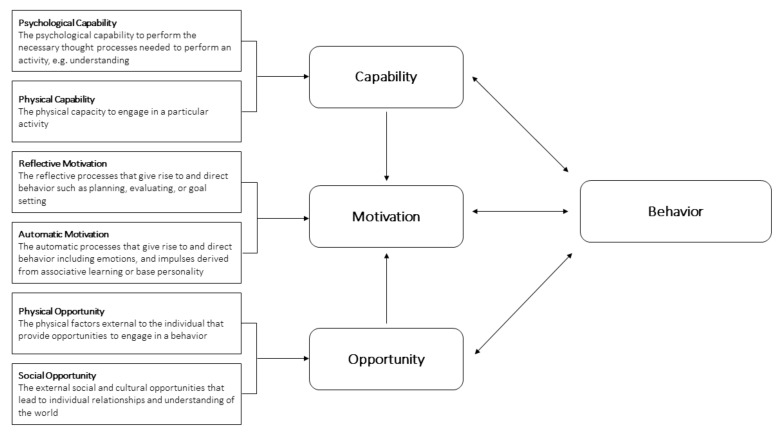
The interaction of the COM-B components and subcomponents within a behavioral system. Adapted from [12].

To date, intervention development has largely neglected systematic processes that link target behaviors (for example, fast food consumption) with the behavioral system (for example, workplace dietary behaviors). To overcome this limitation, and improve our understanding of workplace HLB, we used an overarching framework, namely the capability, opportunity, and motivation of behavior (COM-B) model [12]. The COM-B model captures the individual, interpersonal, and organizational factors that direct behavior and facilitates interventions tailored to behavioral systems. The COM-B model is further broken down into six subcomponents which can, in turn, be influenced by behavior (Figure 1). Application of the model to the needs of our population thus identifies the target subcomponents or change mechanism(s) required to initiate behavior change in a contextualized intervention [12]. Accordingly, we set out to apply the COM-B model to the complex experiences of preconception and pregnant women within their university workplace setting in order to contribute to the development of a tailored intervention. Therefore, the aim of this qualitative study was to use the COM-B model to explore the barriers and enablers to HLB, weight management, and wellbeing needs of working women, specifically during the preconception and pregnancy periods, in an Australian university workplace.

## 2. Materials and Methods

### 2.1. Study Design

This qualitative study was a part of a pragmatic mixed-methods approach to investigate the needs of working women within their university workplace, specifically health and wellbeing during the preconception and pregnancy periods. The researchers considered their respective backgrounds in health science, public health, psychology, and social science in determining the chosen research approach. A pragmatic approach allowed the researchers to derive knowledge from absolute constructs, for example the working environment or workplace policies, and abstract constructs, such as beliefs or identity. The present study utilized focus group (FG) data, informed by the COM-B model, to contribute to a contextualized needs assessment. The needs assessment also includes survey and environmental assessment research and, thus, allowed us to assess the consistency of our findings by triangulating the data (not reported here). The research approach informed how the HLB and wellbeing needs of working women were interpreted and situated the women as experts in their own lives and behaviors. Ethics approval was provided by the Tasmanian Health and Medical Human Research Ethics Committee (H0017313).

### 2.2. Participants and Recruitment

Women employed at a university workplace were eligible to participate, regardless of their age or position. In order to generate a full and detailed understanding of our target population, we purposively sampled participants who may have had a personal or professional interest in our research by manually searching public staff profiles. Following the search, a personalized email invitation was sent to potential FG participants working as health and safety representatives; members of the “Work, Health and Wellbeing Network”; human resources team members; academic staff within health sciences, psychology, and medical research with specializations or interests in general health, midwifery, mental health, chronic disease, or public health; professional staff specializing in student wellbeing, support, counseling, engagement, retention, and advocacy; and staff with a background in digital communication and online platforms, such as social media and mobile-health. Women of all disciplines and backgrounds were also invited via the Work and Wellbeing Survey, previously circulated to all women aged 18–45 at the university, and via advertisements on the staff intranet and in staff newsletters. While these advertisements targeted a wider audience than the personalized emails, FG context was provided by framing the purpose and rationale behind the research (Table S1). Explanatory statements and consent forms were emailed to women who expressed interest in participating. All participants provided informed consent prior to taking part. Although a number of participants were from health or health-related backgrounds, specific roles were not formally recorded in order to maintain participant anonymity.

### 2.3. Procedure

Information was collected from 25 women employees during 5 face-to-face FG sessions across 2 university locations in Tasmania, Australia. Sessions were led by one facilitator (SKM, CMB, or BH) and were guided according to the semi-structured question schedule (Table S2). Questions centered around preconception and pregnancy health, health in the workplace, HLB, barriers and enablers to participation in healthy lifestyle programs or initiatives, and the concept of a workplace health portal for women. Basic demographic information was collected using a 6-item paper questionnaire (Table 1). Field notes were documented by the researchers (SKM, CMB, and BH). Focus groups were conducted between September and November 2018 and lasted approximately 1.5–2 h. Each session was audio recorded and transcribed by a third-party transcription service, with transcripts checked for accuracy by SKM.

### 2.4. Data Analysis

Data analysis was informed by the Collaborative Qualitative Analysis process [13], across six phases: *Phase 1*: *Preliminary organization and planning*. Two researchers (SKM, CAB) met to discuss the underpinning COM-B model [12], research questions, and timelines for data analysis. *Phase 2*: *Open and axial coding*. The same two researchers independently coded and recoded one of the FG transcripts with the assistance of NVivo 12 software [14]. Data analysis was informed by an abductive logic and commenced by structuring codes according to the COM-B subcomponents. COM-B subcomponents were then populated with general codes and conceptual labels deriving from participant data and field notes. The researchers met to discuss the initial coding and resolve any discrepancies. *Phase 3: Development of a preliminary codebook.* Following several discussions, the first author (SKM) generated a preliminary codebook. *Phase 4: Pilot testing the codebook.* The preliminary codebook was reviewed by the second author (CAB) before being applied to the rest of the transcripts by the first author. The codebook was then reviewed by two researchers (CAB, BH) before being finalized (Table S3). *Phase 5: Final coding process*. All coded data was reviewed and discussed by three researchers (SKM, CAB, BH). *Phase 6: Finalize the themes.* Themes and subthemes were then constructed iteratively and collaboratively with the wider research team to align with the aims of this study; however, prior researcher knowledge, assumptions, and assessment of the Work and Wellbeing Survey may also have influenced this process.

## 3. Results

### 3.1. Participants

The mean age of the 25 women was 44.1 years (Table 1). Sixteen women had children (64%) and 16 women were engaged in full-time work (64%). Women held diverse views regarding the definition of the ‘preconception’ life stage. Many women described ‘preconception’ as a period of pregnancy preparation and planning. However, others conceded that this description excluded women without pregnancy intent. The perceived duration of the preconception period varied considerably. Women reported that the length of preconception could be impacted by age or parity. Some women felt it was a lifespan occurrence, moving from the time a woman’s ovaries are set in utero until a woman reaches menopause. Other women thought the preconception period related to a woman’s capacity to become pregnant and, therefore, started at menarche. Women also spoke of how the preconception period could be of importance for women to organize their work–life balance, leave entitlements, finances, health insurance, supplementation and vaccinations, access health care, address bad habits (eating, drinking, or smoking), improve their knowledge around pregnancy (books or courses), improve their physical environment (food landscape or exposure to cleaning chemicals), prepare psychologically to become a mother, and improve their HLB.

### 3.2. Themes

**Figure 2:**
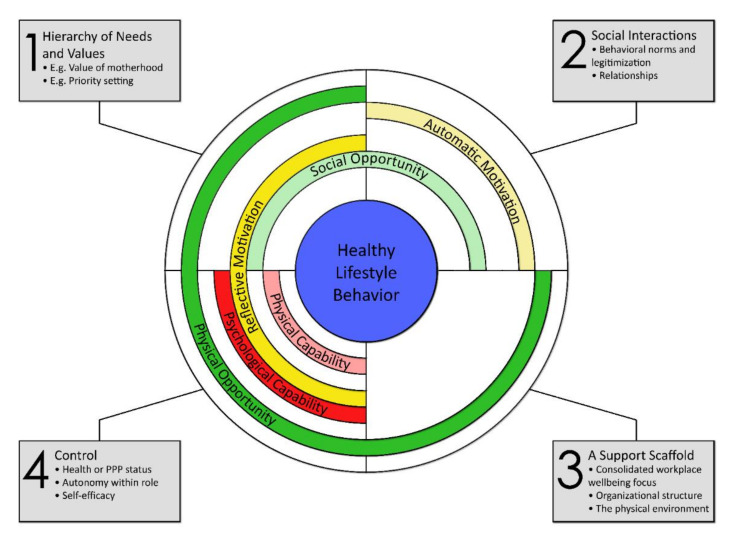
COM-B subcomponents organized according to the four overarching themes. Key: PPP = Preconception, Pregnancy, or Postpartum.

Barriers and enablers to HLB and/or wellbeing were identified across all six COM-B subcomponents and were developed into four overarching themes (Figure 2).

#### 3.2.1. Theme 1: Hierarchy of Needs and Values

The hierarchy of needs and values theme explored how women experience and assign value to their needs, with a particular emphasis on the challenges of being a woman of reproductive age. Hierarchy of needs and values aligned with the physical opportunity, social opportunity, and reflective motivation COM-B subcomponents. Women spoke of how they assigned value to their own behaviors and priorities, for example, high workloads during busy periods overshadowed health or wellbeing needs for some women.

*There is the time problem… sometimes, depending on the time of the year, if I have deadlines, I don’t have time to go to the gym.*

(FG5)

Women discussed how their work demands vied for priority over caring responsibilities, other life demands, and HLB. The value assigned to these priorities fluctuated according to life circumstances and experiences of pregnancy and parenting at work. One woman described how her role consisted of “lots of urgent, high stress” work and expressed concern over how this could “jeopardize a baby”, should she become pregnant. Further, some women noted that work obligations left them too tired to engage in their personal lives or HLB.

*I totally get the message that health is also not optional, but the reality is if there’s ten things on the list, and I can only get through seven of them, I have to…prioritize these other things.*

(FG5)

Women tended to feel that their mental health needs and wellbeing were valued less than the needs of the university.

*It made it even worse because I was, like, so upset and I got told ‘be strong for your team’. It’s like, what? So, I’m not allowed to be upset? And that was pretty much the message I got was ‘be really strong’.*

(FG2)

Many workplace opportunities to provide employee support and meaningful interaction were supplanted by “box ticking” activities, i.e., the minimum legislative requirements. In keeping with these observations, women perceived that staff and students were treated differently and felt that student wellbeing was prioritized over staff wellbeing. Women spoke of a dual standard, where it was more culturally acceptable for students to be pregnant or to bring their young children on campus.

*I think that with students you visually see them… like those two women who can have their babies at University and still study, like that is a fantastic thing visually, but I don’t think I could, if I had a baby, I couldn’t bring my baby to work.*

(FG1)

Although women reflected that short-term contracts and casualization are common in the university sector, they also said job uncertainty made them feel undervalued and unappreciated for their depth of experience and skill. One woman spoke of how insecure work stopped her from “living her life”.

*Also, in your job… like I love doing my job well, but… my current contract’s over in six months and I can already feel myself going, ‘I don’t give a shit about that anymore’, whatever it is, because I’m not going to be there, you know, pushing it anymore… But I don’t know why the university don’t value, you know, staff like myself a little bit more.*

(FG2)

Some women discussed the distress and uncertainty that accompanies job insecurity. They spoke of how it limited their ability to make decisions relating to family planning, preconception, and maternal health. Women questioned whether they could afford basic items, for example a sewing machine, if their contract were not renewed.

Transitioning to motherhood seemed to symbolize a loss of status for some women, as they moved from their work role to their new domestic role. This decrease in status was reinforced where women returned to work after taking parental leave and experienced a loss of resources, such as office space, when electing to reduce their working hours.

*When they come back from maternity leave…they consequently lose their office and they go to a shared environment, where previously they had their own office with their own things. So, there’s a disadvantage there in even having a baby, because you are coming back with less than what you started with…it’s just what it says…*

(FG5)

Within the workplace setting, some women expressed concern that pregnancy status may lead to negative colleague perceptions regarding capability. For other women, simply being of childbearing age was seen as undesirable and presented a major obstacle to obtaining employment.

*I’ve worked in many different workplaces, like a lot of male-dominated workplaces, and the comments, not necessarily directed at me, but directed at maternity leave in general here have been, like I was so shocked about just the comments, like there was an academic that basically said he would not employ… a woman of child-bearing age.*

(FG1)

Women discussed how similar social and physical barriers to wellbeing were not shared by men, when combining having children with furthering their career.

*You do get the sense that women carry that unfair burden of the leave stigma, I mean there’s also a man at work somewhere who’s a father to a baby, who’s part of that conception, part of that gestation process and that birth process, whose career is not touched, you know, and it’s not fair is it?*

(FG1)

In summary, women spoke of how they assigned priority to their non-negotiable needs, i.e., work and caring responsibilities, rather than ‘optional’ health and wellbeing-promoting behaviors. Women also felt that there was a social hierarchy within the university, where the needs of students and male colleagues were thought to be of greater value. The perceived low value of the women was exemplified through instances of job insecurity, experiences of transitioning to motherhood, and workplace attitudes towards the mental health of staff.

#### 3.2.2. Theme 2: Social Interactions

The social interactions theme identified key social influences that could either facilitate or act as a barrier to women’s HLB or wellbeing. The theme is divided into two subthemes which align with the social opportunity and automatic motivation COM-B subcomponents.

##### Relationships

Women identified workplaces as important facilitators of social connection. They discussed how their work relationships supported opportunities for engaging in workplace wellbeing activities, motivated weight loss, and encouraged participation in group competitions, for example the “10,000 Steps Program”.

*Members of the team go out… They were walking three to four times a week, even [if] it’s just, like, 45 min to an hour, particularly if the weather was good…*

(FG2)

When asked about workplace wellbeing activity facilitators, women responded that they were more likely to engage if they knew other participants; the social aspect of the group wellbeing activities seemed to be as important as the participation in these activities. Women also reflected how having someone to ‘take the initiative’ (workplace wellbeing champions) supported opportunities for health promotion, including facilitating access to healthier food at meetings and encouraging participation in walking meetings, standing desk usage, and engagement in wellbeing activities. Further, work relationships enabled women to foster social capital and belonging. Women spoke of how some departments regularly took morning tea or lunch together, celebrated birthdays, or attended start of semester drinks. Women said that colleagues were often the driving force that enabled them to take breaks from work or go for a walk.

*They really understand connectivity… like every month they celebrate somebody’s birthday. They’ll celebrate…they’ll connect, they come out and have lunch together…those kinds of things.*

(FG4)

Women discussed learnings from their female colleagues about workplace pregnancy or parenting entitlements, such as leave entitlements or conference attendance support; shared information based on lived experience of the preconception, pregnancy, or postpartum periods; and received help, particularly in the absence of a supportive work culture or appropriate physical supports.

*It was really tricky early on…to find somewhere to, to breastfeed on this campus, and my success in continuing to breastfeed was because I had a boss who…as we got to know each other a bit better… She had a tiny office, and we would sit back-to-back, while I was doing it.*

(FG3)

In contrast, women also identified how their manager or colleagues could make them feel burdensome, when balancing their caring responsibilities. Consequently, women spoke of a massive variation in experiences across the organization, attributable to the relationships women had with their supervisors and colleagues. Women who had positive experiences with their transition to pregnancy and parenting at the university acknowledged that they were likely in the minority.

*I think there’s an incredible variety of experiences of this here, there’s such a vast organization and there are so many decision makers and so much depends on your immediate supervisor. And I hear stories that are really positive, and you just think, ‘that’s fantastic’, and then you hear other stories and you just think, ‘What decade are we in?’*

(FG5)

Ultimately, women described their colleagues as a source of motivation to engage in healthy lifestyle behaviors, take breaks from work, and learn about parenting- or pregnancy-related workplace supports. Conversely, women acknowledged that managers or colleagues could also make women with children feel like a burden, when balancing their caring responsibilities.

##### Behavioral Norms and Legitimization of Health Behaviors

Women discussed a genuine need for workplace managers and peers to embody the HLB and wellbeing focus that were frequently promoted at an organizational level. That is, they needed to observe their colleagues and managers adopting the behaviors themselves. Women said this would create a legitimizing effect and, thus, would enable and empower them to participate, and prioritize their health.

*I do have a boss and the next boss up as well who are very, like, big on lunch breaks and will… be seen even when they’re busy going and taking a lunch break and they will encourage you to take a lunch break.*

(FG3)

However, women in management positions did acknowledge that although they encouraged their team to take breaks or exercise, some did not partake themselves, citing workloads or resourcing (for example, single-point dependencies) as main barriers.

*I’m in a middle management position and I would say that I’m a really bad example to my staff… like most people definitely get a lunch, a lot of people are able to go for walks and things like that. So, I would encourage and support that… But yeah, I work all day, I go home and…still work and I work on the weekends to do…my job. But that’s life.*

(FG2)

On the other hand, flexibility was highly valued among participants. It seemed to be accessible to most women and there appeared to be a cultural norm around flexibility at work. Women with children seemed to use flexible working arrangements to coordinate caring and work responsibilities, whereas women without children were better placed to integrate physical activity into their workday.

*[It] can be a very flexible working environment as well, like working around your children and there’s a lot of people where I work, there’s hardly anyone that actually works nine to five, five days a week. They’re all starting early, finishing early or staying late, or just not working that day or doing all sorts of hours or working from home and school pickups and it’s… really supportive.*

(FG2)

Workplace cultural norms also served to highlight receptiveness to pregnancy in the workplace. Women looked to their pregnant colleagues to inform their expectations around workplace pregnancy or parenting experiences. However, one woman spoke of how she was unable to form clear expectations in the absence of viable role models in her department.

*There was one girl who was pregnant a few years ago. I don’t really know… Probably not anyone who’s in a management role like me, rather [those who] already have had children or a lot of them are males to be honest.*

(FG3)

Conversely, in female-dominated areas, pregnancy was perceived as a recurring inconvenience, where replacement staff were continuously sought to fill in for women taking parental leave. Consequently, although women getting pregnant and having children was very commonplace, there appeared to be a negative cultural undercurrent to pregnancy in the workplace.

*We had probably a higher number of women and young women, so it was always a bit of a running…you know, oh gosh, trying to replace the person that’s having the baby, so rather than see that as a problem because they’re like, oh my God, like now you know, who’s going to do that job?*

(FG2)

To summarize, although participants felt it was important that management and colleagues exemplified health-promoting behaviors at work, those in management positions often felt unable to do this due to resourcing or workload issues. Normalization of parenting and pregnancy at work, for example by providing ready access to flexible working arrangements, was important for helping women set expectations for subsequent pregnancy and parenting experiences at work.

#### 3.2.3. Theme 3: A Support Scaffold

A support scaffold describes the importance of physical influences, including policies or organization-level supports, on women’s HLB and wellbeing at the workplace. The theme is divided into three subthemes, which align with the physical opportunity COM-B subcomponent.

##### Consolidated Workplace Wellbeing Focus

Many women discussed the need for a strategic and genuine focus on staff wellbeing, integrated across all facets of workplace operations. Several women mentioned that although there were many physical opportunities for HLB at work, activities seemed to be organized in an “ad-hoc” or uncoordinated manner that was not sustained over time.

*[The workplace] does a lot of ‘ad-hockery’, so you have things that [are] offered and it’s someone who’s got passionate and made it happen. And you don’t know if it’s going to carry on, it’s just…you don’t know if it’s consistent.*

(FG5)

Women stated that healthy lifestyle or wellbeing activities were often poorly advertised and that formal information dissemination pathways were absent or unnecessarily complicated. Similar confusion existed around women’s leave and contract entitlements, with many women being unclear as to where to source such information.

*Yeah, so I think it’s probably something that needs to be broadcast a bit more and there’s a lot of things, like, that I think on this campus that are just not well organized and communicated.*

(FG4)

It was evident that women experienced disparate access to workplace wellbeing opportunities relative to workplace location (including healthy lifestyle activities, office space, standing desks, shower and change areas, kitchen and lunch facilities, and private spaces), affordability (including cost and salary sacrificing availability), relevance, choice, regularity, and quality. Availability of staff resources to share workloads or provide ‘cover’, also influenced whether women performed HLB at work. Additionally, although most women felt that their workplace had a responsibility to not negatively impact their health, many initiatives seemed to be individually driven by workplace wellbeing champions. One woman noted there were no appointed staff positions to coordinate health and wellbeing for employees. There seemed to be an expectation that individuals would step in to fill this gap.

*It comes down to the… whose responsibility is it though? Like we don’t have a health and wellbeing full-time staff member that runs all these things…*

(FG4)

Women also highlighted how barriers to physical wellbeing opportunities extended to a lack of policy focus and coordinated action plans around employee wellbeing. Women remarked that there were no explicit policies around wellbeing and felt policy development was typically ‘reactive’ rather than ‘proactive’. For example, women spoke of a recently implemented campus-wide smoking ban that sought to align the workplace with current legislation. The women expressed disappointment regarding the absence of complementary smoking cessation programs, enforcement, compliance, and signage. The disjointed approach to wellbeing created a foundation for negative social culture to become established, thus impacting wellbeing opportunities. Women felt the workplace culture was not ‘people-centered’ and gave rise to excessive workloads and increased stress levels.

*We are working in a culture, in an environment that hasn’t been people-centered for a long time and stress is just considered a normal part of doing business, and people’s stress levels… there’s a reason they stopped doing the staff surveys. They don’t want to know…*

(FG5)

One woman suggested that the issues around culture and organizational commitment to wellbeing also had a flow-on effect for women who were pregnant or already parents.

*If you are seeing pregnancy or parenting as a challenging situation to manage at work, then that says something about the place that you’re working in. If you are having to figure out how I can make this incredible life event have the minimal impact on my workplace and my career, the way I do my job…*

(FG5)

Overall, women observed that there was varying access to workplace wellbeing opportunities, such as standing desks and showers, and that employee health and wellbeing were typically uncoordinated and individually driven.

##### Organizational Structure

Some women discussed their lack of opportunity to participate in workplace wellbeing activities offered outside their immediate department or area (i.e., silo). There seemed to be a quasi-physical barrier produced by ‘siloing’ that deterred or prevented women from outside the silo availing of healthy lifestyle and wellbeing opportunities. Women spoke of how this barrier was reinforced by their perceived lack of belonging within other work areas, a lack of positive social interactions during silo ‘infiltration’, resource guarding, segregated department mailing lists, and social cliques. These perceptions also accentuated how healthy lifestyle opportunities and wellbeing supports were not universally available to all employees within their immediate silo.

*Yeah, having facilities and feeling like you can use them away from your desk because… I’m not a part of the school, I’m employed centrally and sometimes when you look into the team and you, you get funny looks…*

(FG1)

In short, the separation of the workplace into different buildings and departments created a real barrier to social and workplace wellbeing opportunities.

##### The Physical Environment

Women felt the physical environment impacted their HLB and wellbeing opportunities. Women were critical of the food landscape at their workplace. Healthy options had declined over time and were often unavailable, particularly for women who were time-poor or preconception women trying to lose weight. Discretionary foods were typically provided at meetings and work functions, and as office snacks. Food options at cafés were lacking in nutritional information; were not conducive to ethical, sustainable (i.e., local), or culturally appropriate eating; and many ‘healthier’ options were often accompanied by fried foods. One woman remarked that while she did not bring unhealthy foods into her home, it was impossible to avoid them at work.

*I think the food offerings on this campus are abysmal, have been for many years, getting worse and they don’t support healthy eating. They make it very hard to eat… Especially if you are just pushed for time and you just need a quick something, it’s very hard to choose healthy if you are grabbing something and going back to your desk. They need to make it much easier.*

(FG5)

Further, some women felt that there were few spaces to sit outside and eat lunch away from their desk, and that tearoom fridges were too small to store homemade staff lunches. Some women mentioned that food cost and choice varied according to their workplace location, although others reflected that future campus redevelopment and relocation may provide more alternatives.

*These little bar fridges, everyone brings their lunch and they’re packed in there, and it’s not really accommodated in some areas for people to all bring their lunch and have a fridge.*

(FG4)

Incorporation of active design elements, including stairs access, campus layout or size, and campus location allowed some women to benefit from incidental exercise as a part of their typical workday. However, access to this physical opportunity was unequal among participants. Women discussed how certain work settings, for example a city-center versus suburb location, could facilitate variable opportunities for active transport, exercise, and community engagement.

*I have to move around a bit as my stakeholders are located in many places and this campus being on a hill, I get to walk up that hill quite a lot and stairs, three flights.*

(FG1)

Many women said physical workplace parenting supports were inconsistent or absent (including parenting rooms, childcare, parental leave toolkits, contact days during parental leave, private office space, and conference support for academics with caring responsibilities). Further, information or policies to guide or protect preconception and pregnant women within laboratory settings was notably lacking, thus potentiating risk to women during this life stage.

*There’s nothing available, you have to physically talk to someone. And sometimes you won’t want to talk to your supervisor to say, ‘hey, I’m thinking about becoming pregnant. Is there somewhere that I shouldn’t be going in the lab?’*

(FG2)

In brief, participants critiqued the lack of healthy food options, social spaces for staff to sit and eat, and the inconsistency of access to physical parenting supports (e.g., childcare) at their workplace. Women observed how building design (e.g., stairs access) and their workplace setting (e.g., suburb location) could facilitate incidental exercise.

#### 3.2.4. Theme 4: Control

The final theme, control, related to factors that impacted women’s ability to manage their HLB, weight, or wellbeing needs, such as health status or self-efficacy. The theme branched into three subthemes and aligns with four of the six COM-B subcomponents: physical opportunity, physical capability, psychological capability, and reflective motivation.

##### Health or Preconception, Pregnancy, or Postpartum (PPP) Status

Some women spoke of how their health or PPP status impacted their wellbeing, preconception preparation, or ability to engage in HLB. Health status, including injury, sickness during pregnancy, autoimmunity, and age acted as barriers to health, wellbeing, and/or weight maintenance. For example, one woman recounted how an injury, combined with dietary behaviors, led to weight gain.

*I had a dodgy ankle and I tend to like food for comfort, and I wasn’t able to do as much exercise. Um, so you know, put a bit of weight on and formed lots of bad habits like eating chocolate after meals and eating chocolate all day long really.*

(FG2)

With regards to weight, participants indicated that achievement of preconception weight loss could improve IVF success, offspring outcomes, or alleviate chronic conditions (e.g., thyroid conditions), albeit prolonging the preconception period. Perhaps worryingly, given the tendency of those in academia to delay pregnancy, older first-time mothers felt they had less time to improve their health behaviors or health status during the preconception or interconception periods.

*For some people, if it is hard to get pregnant, the main thing you worry about is whether it works or not… But yeah, the idea of waiting until you are healthier, I hadn’t been in a position to think of that and I think it’s great that you can.*

(FG3)

In sum, factors such as health status (e.g., injury) and age acted as barriers to health, wellbeing, and/or weight maintenance of women. While participants felt that weight loss could improve certain outcomes (e.g., offspring outcomes), some felt older mothers were under greater time pressure to conceive and, therefore, felt unable to make positive health behavior changes.

##### Autonomy Within Work Role

Women discussed how modifiable attributes of their work role, such as having control over their work scheduling, allowed them to accommodate their non-work needs, including taking breaks, having lunch, exercising, caring responsibilities, prioritizing mental health, and attending appointments (e.g., pregnancy-related) or events. However, women in academic roles also felt control over how and when their classes were organized had been replaced by top-down directives. This loss of autonomy prevented several women from engaging in physical activity at work and for some women, for example those with small children, this could be their only opportunity to incorporate exercise into their day.

*So many times, you can be doing two-hour practical tutorials in the… lab, four of them in a row, back-to-back…You’re supposed to have 10 min, but of course all the students were desperate to talk to you.*

(FG4)

Some women indicated that access to flexibility entitlements (including flexible working arrangements, working from home, and reduced hours) depended on manager discretion. Others discussed the prevailing notion that flexibility was only required for parents or women with children. Further, some women remarked how working in a reduced-hours position would likely still result in a full-time workload.

*But someone who I know does work kind of reduced hours and is in a position which is kind of a similar level to mine. And she kind of leaves early, which is a bad way of phrasing it, but… I feel like you’d just end up doing the same amount of work anyway.*

(FG3)

Part-time or flexible working also instilled guilt in some women when day-to-day work activities were scheduled outside their working hours.

*So, all our committee meetings are on a Friday and I’m not working on a Friday at the moment, so I’m on committees, but it’s meaningless because I can’t come the meetings… You’re an apology, but I actually feel like I’m apologizing for not being there. I feel bad. I’m letting them down, how can I be on a committee and not turn up? And I’m not paid to be there.*

(FG3)

In addition, part-time or flexible worker status hindered some women’s self-determination to attend the gym, take their lunch, or participate in workplace wellbeing activities during work time. These women felt they could not ‘ask for more’ from their employer.

*In a way I already feel so compromised working part time and not just working part time but consistently working just slightly shorter hours on every day than I really should or would like to… It feels like I can’t ask anything more of [my workplace] like that, like I’m squeezing from them as much as I can simply by coming back part time and working those shorter hours. So, the idea for example, of going to the gym at lunch time… It’s almost incomprehensible to me because I work through lunch because I’m going to be leaving at quarter to four to pick up the kids and I might spend 10 min eating a sandwich…*

(FG3)

Women also discussed how core aspects of work roles could present a challenge, for example front-facing reception roles or roles without staff cover limit one’s ability to manage sick children or engage in HLB during the workday. Also, women in low-autonomy roles may not have the same access to flexibility as other staff.

*There are people, like, who are in relatively low, like, frontline roles like call centers or reception type roles where if they go out for the day, like kid gets sick you have to go pick them up, it actually does cause a challenge. It’s a management challenge…*

(FG5)

Many women expressed concern regarding the increasingly sedentary nature of their work. It was felt that academic staff frequently skipped morning tea breaks, typically ate at their desks, and had less opportunities to partake in incidental exercise or in-person contact with colleagues following the introduction of communications technology such as Skype.

*But it’s more than food for me, it’s also the sedentary nature of my job, it’s extremely sedentary and more so every year as we become more efficient and the job requires more just sitting at your desk and powering through the work, less opportunity to move during the day. That’s a really a big thing for me, is that the work itself is unhealthy.*

(FG5)

Additionally, several women in academic roles felt that academic culture was not conducive to parenting responsibilities, given the high workloads and irregular hours. It seemed that excessive working hours were the norm within academia, and this was coupled with the belief that many hours were needed to advance your career. Although many women felt heavy workloads encroached on their personal time, this extra work was never acknowledged within the workplace to be unusual as to do so would concede the work was excessive and, thus, capable of impacting wellbeing. One woman recounted a former colleague’s frustrations:

*She said, ‘the reason I quit [was] because they used to message me every single night asking me to look at things at nine, ten o’clock at night’. And she goes, ‘I wanted to be at home with my kids’ or just, ‘that was my downtime’.*

(FG2)

Summing up, several women remarked that modifiable aspects of their work role (e.g., flexible working arrangements or part-time status) acted as a barrier to participation in workplace wellbeing activities, or even taking a break. Further, women felt they had little ability to improve core aspects of their work roles (e.g., sedentary office roles or academic culture).

##### Self-Efficacy

Finally, women discussed how their confidence to perform HLB or attain goals was affected by a number of barriers including: (1) not being aware of essential information or knowing where to access it, such as parental leave entitlements; (2) not setting goals or timeframes in relation to the PPP periods (for example, there is ‘no right time’ to fall pregnant), thus precluding improvements to HLB; (3) the physical and social workplace environment, including ease of access to unhealthy foods like chocolates in the office or stigma associated with mental health support; (4) long workdays and sedentary roles; (5) having alternative priorities not focused on health, such as work responsibilities; (6) experiencing failure when trying to meet physical activity or weight goals; and (7) believing that lots of time is required to perform physical activity behaviors or engage with health technology, such as apps.

*I definitely think sometimes, as someone who would’ve liked to have kids at some point, I do feel like a real pressure around the fact…I’ve probably got more time to do things in my life right now… I feel quite busy as it is… I have recently gotten more back into exercising and I do it before work because I find I don’t have time during the day and I can just talk myself out of it if I have the whole day. It’s, like, five reasons by the end of the day why I shouldn’t go, and it just makes sense to go straight home and sit on the couch.*

(FG1)

Conversely, women identified a number of facilitators to self-efficacy including: (1) having the capacity to identify health concerns requiring improvement and potential solutions, for example, overcoming workplace sedentary behaviors with a standing desk; (2) perceiving that activities or behaviors are valuable, for example mental health is as important as physical activity or diet for health and wellbeing; (3) social support and encouragement; (4) goal setting and tracking (technology such as apps and fitness watches can support this); (5) practicing mindfulness; (6) scheduling breaks into the workday; (7) breaking physical activity down into smaller, manageable blocks to accommodate work; (8) prior positive and successful experiences, for example, asserting entitlement to work flexibly during a second pregnancy; (9) planning and preparation, including bringing your own lunch or snacks to work (especially if healthy options are unavailable); and (10) believing that weight and health behaviors require self-management.

*I brought my gym to my office because I have room, my office just has three desks and I’m in the corner and I put everything over there for my exercise so that bench is not huge… And I just make myself go there every two hours and do exercise.”*

(FG2)

In summary, barriers to women performing HLB or achieving their goals included the physical and social working environment, having alternative priorities not related to health, and failing to meet physical activity or weight goals. Facilitators to HLB or attending to pregnancy or parenting needs included being able to identify health concerns and potential solutions, goals setting and tracking, and having prior successful experiences.

## 4. Discussion

This study drew on the insight and experience of women of all ages to illustrate the experiences and challenges of working women of reproductive age, with respect to their health and wellbeing specific to the preconception and pregnancy periods at their university workplace. By highlighting the multi-level behavioral influences on working women of reproductive age, application of the COM-B model has helped identify the shape and complexities of behavioral systems, relative to context and individual needs. The barriers and facilitators to the HLB and wellbeing needs of the women included: high workloads, caring responsibilities, workplace support and understanding, job insecurity, discrimination, colleague relationships, workplace norms and academic culture, policy, information dissemination and availability, siloed departments, the food landscape, active design and workplace setting, age and health status, control over work scheduling, access to flexibility, part-time or flexible worker status, and self-efficacy.

In the current study, themes mapped onto all six COM-B subcomponents; however, opportunity (i.e., social and physical opportunity) seemed to have greater relevance to the participants (Figure 2). This indicates that understanding opportunities (i.e., contexts external to the individual) may be key to deconstructing and clarifying workplace behavioral influences on women’s HLB. Thus, subsequent interventions should incorporate these components when devising contextualized strategies for behavior change. The relative importance of ‘opportunity’ is somewhat contrary to other qualitative studies conducted with preconception and pregnant women, which often focus on individual capacity, for example knowledge, rather than setting-specific opportunities [15,16]. However, this difference may relate to our study question schedule that directed women to concentrate on workplace-specific factors. We also applied a pragmatic approach that was practical, and stakeholder focused, in order to position women’s beliefs as tangible constructs and, thus, align with opportunity rather than capability. Further, women’s experiences of workplace culture may have caused them to assign greater weight to external factors impacting their preconception, pregnancy, or parenting needs rather than their capacity for HLB. Below, we discuss our main findings with respect to capability, opportunity, and motivation in order to understand the key factors to improve the health and wellbeing of these women in the workplace setting.

### 4.1. Capability

While capability was less prominent in the themes, relevant discussions tended to center on women’s capacity for physical activity. Women placed comparatively little emphasis on their capability to engage in healthy eating or other wellbeing-related behaviors. Women may have felt that healthy eating or wellbeing-related behaviors did not require the same skills or ability as physical activity participation, for example fitness levels. We found that women wanted workplace healthy lifestyle opportunities that corresponded with their individual capability levels and tailored to their fitness, health, or pregnancy status. Recently updated physical activity guidelines have stressed the importance of regular movement for pregnant and postpartum women (unless contraindicated) within their work, home, pedagogical, and community settings [17].

Previous lifestyle interventions to improve the health and weight outcomes of pregnant women with overweight and obesity in antenatal settings have been hindered by women’s pre-existing health conditions or low preconception fitness [18]. Conversely, a recent systematic review highlighted that workplace physical activity interventions can benefit weight and physical activity outcomes for working women [11]. However, given the low adherence to physical activity recommendations among women of childbearing age [2,19], it is clear that interventions must focus on enhancing women’s capacity for physical activity during preconception. Further, our findings revealed that women perceived that losing weight may alleviate chronic conditions. To support HLB, workplaces should facilitate equitable access according to ability and health status, including for women with chronic illness, disabilities, and those recovering from the birthing process. This provision would provide women with the autonomy to improve HLB into pregnancy and beyond.

### 4.2. Opportunity

Physical and social opportunity were prominently represented across the themes. Regarding opportunities for wellbeing and engagement in HLB, women discussed how lack of support and autonomy were important barriers within the workplace. Women needed greater vocalization and demonstration of workplace support for their parenting, pregnancy, and wellbeing needs to enable access and engagement with health and wellbeing opportunities. Consistent with other studies conducted at university and hospital workplace settings, participants repeatedly spoke of the importance of top-down support for employee wellbeing [20,21]. Further, the presence of workplace wellbeing programs can communicate employer investment in employee health and provide employees with the ‘excuse’ to prioritize their health [21]. It was clear from our study, however, that many participants felt that organization-level needs supplanted the wellbeing needs of employees. Indeed, within the workplace, there was an over-reliance on individuals to fill the gaps in access and support, which typically led to wellbeing opportunities not being sustained over time. This over-reliance also resulted in disparate and inconsistent access across the organization.

Previous research has iterated the need to consider whether the effort to improve employee HLB is of greater benefit to workers than addressing workplace factors that cause distress, for example, negative workplace culture [21]. Certainly, a combination of both strategies may have the greatest impact on health outcomes. However, this observation suggests that employee health promotion and endorsement are not limited to availability and access. We know that psychological wellbeing during pregnancy is associated with improved HLB [22] and it is evident from our study that the influences of workplace opportunities on working women’s wellbeing needs are complex and far reaching.

### 4.3. Motivation

We found that women frequently downgraded their health and wellbeing needs to prioritize their work or caring responsibilities, which resulted in reduced motivation to achieve personal health outcomes. This hierarchy of needs and values may derive from prevailing environmental and social cues that project a lesser value on the needs of women [23]. Further, our findings indicate this ‘lesser value’ was reinforced within the workplace setting, where women’s fulfillment of caring responsibilities or wellbeing needs required frequent navigation of workplace norms, processes, and levels of acceptance. Similar health priorities have been found in an office-based study, whereby workers gave precedence to getting work done over reducing their sedentary behavior [24]. It has been observed that workplace demands may not be conducive to following prevailing healthy pregnancy guidelines and can, effectively, obligate women to deemphasize their pregnancy-related needs at work [25].

Information on ‘the consequences of behavior’ and women’s beliefs about their capability and mindset could be important facilitators of HLB during pregnancy [16,26]. Interventionists have often attempted to capitalize on pregnant women’s enhanced motivations [27]. However, the short duration of pregnancy and competing needs can make behavior change difficult during this transient life stage [28]. Additionally, current evidence suggests that pregnancy intentions are not associated with improved preconception diet and physical activity behaviors, despite the importance of healthy lifestyle behaviors before pregnancy [29,30]. However, our findings suggest that women feel health priorities are more reasonable and warrant workplace support during pregnancy, thus sanctioning a greater focus on self-care. Altogether, this would suggest that greater education and workplace support for preconception health and wellbeing may motivate and ‘authorize’ women to improve their HLB before pregnancy.

### 4.4. Strengths and Limitations

Strengths of this study include applying the COM-B model, an overarching framework used to conceptualize behavioral influences. Abductive analysis ensured that key concepts and participant experiences were explored. However, limitations include the fact that we did not solely recruit women of reproductive age and this may be construed that the participants did not have the authority to speak about the challenges faced by this population. However, we would argue that the inclusion of a diverse age range lends credibility and richness of experience from women who have lived through this life stage. Member checking was not conducted to improve the validation of participant responses. However, as the current study is part of a larger needs assessment for the target population, this project-level limitation has been minimized through data triangulation. Lastly, study participants were not recruited from all campus locations operated by the university.

### 4.5. Future Directions

The importance of exploring the contextual barriers and enablers unique to women during the preconception and pregnancy periods is clear. Our findings suggest that future workplace interventions should explore women’s needs using holistic frameworks, such as the COM-B model, to expand understanding and application of behavior change techniques. However, subsequent steps should apply the current findings to increase intervention capacity for success. Specifically, the subsequent multi-pronged intervention must seek to improve: (1) education and support for preconception healthy lifestyle behaviors (capability and opportunity); (2) equity of access to opportunities, according to individual capacity (capability and opportunity); (3) top-down coordination and support of healthy lifestyle activities and programs (opportunity); and (4) the atypical factors impacting the physical, emotional, psychological, or social wellbeing of working women, for example discrimination, parenting supports, workplace culture, and policy (opportunity and motivation).

Finally, while this study investigated the preconception and pregnancy periods, many women discussed their needs in relation to postpartum or parenting. Evidently, these needs were also an important part of women’s experiences during this life stage and highly applicable to the workplace. Thus, intervention development should broaden its scope and increase its relevance, according to the needs of working women.

## 5. Conclusions

Working women can experience workplace-specific barriers and enablers to their HLB, wellbeing, and weight maintenance during the preconception and pregnancy periods. Our findings highlight the importance of conducting a contextualized investigation into the disparate needs of working women of reproductive age, prior to intervention development. A multi-pronged approach to improve women’s capability, opportunity, and motivation to engage in HLB—targeting aspects such as preconception healthy lifestyle behaviors, workplace endorsement of preconception and pregnancy health, and facilitation of equitable access to wellbeing opportunities—is likely to have the greatest impact on women in this setting.

## Figures and Tables

**Table 1:** Demographic Information of Participants (*N* = 25).

Variable	Participants
Age, mean (range), years	44.1 (27–62)
Children, n (%)	
Yes	16 (64)
No	9 (36)
Number of children	
Mean (range)	1.3 (0–4)
Median	2
Workplace role, n (%)	
Professional *	14 (56)
Academic	9 (36)
Other	2 (8)
Employment fraction, n (%)	
Full time	16 (64)
Part time	9 (36)
Employment status, n (%)	
Continuing ^†^	11 (44)
Fixed term ^‡^	5 (20)
Casual ^§^	2 (8)
Not stated	7 (28)
Work Location, n (%)	
Hobart Area (Focus Groups 1–3)	13 (52)
Launceston Area (Focus Groups 4–5)	12 (48)

* Professional staff—administrative staff in higher education settings. ^†^ Continuing—ongoing, continuous employment with no defined end. ^‡^ Fixed term—employment for a specific duration, e.g., 2 years. ^§^ Casual—employment without guaranteed hours or duration of employment. Casual employees do not benefit from basic employee entitlements, including paid sick leave or annual leave.

## Data Availability

The data presented in this study are not publicly available in order to protect the privacy of the participants.

## Supplementary Materials

The following are available online at https://www.mdpi.com/article/10.3390/ijerph18084154/s1, Table S1: Focus Group Advertisement for Hobart Area, Table S2: Focus Group Question Schedule, Table S3: Codebook Informed By the COM-B Model.
