# Supplementary material for: Workplace Healthy Lifestyle Determinants and Wellbeing Needs across the Preconception and Pregnancy Periods: A Qualitative Study Informed by the COM-B Model
Source: Int J Environ Res Public Health. 2021 Apr 14;18(8):4154. doi: 10.3390/ijerph18084154 (PMC8070920; doi:10.3390/ijerph18084154)
Supplement: Supplementary file 1 [file ijerph-18-04154-s001.pdf]

Supplementary Material

Table S1. Focus Group Advertisement for Hobart Area

**What:** Work and Wellbeing Focus Group Sessions<br><br>**When:** Thursday 6th September<br><br>**Times**: 09.45-11.30; 12.00-13.45 and 14.00-15.45<br><br>**Where:** [Workplace 1, Hobart Area]<br><br>**All female academic and professional staff** are invited to attend the Work and Wellbeing Focus Group sessions which are aimed at shaping the design and content of the HIPPP (Health in Preconception, Pregnancy and Postpartum) Portal. The function of the Portal is to provide a free, easy-to-use health resource containing reliable information; access to apps and social media; innovative health strategies; current research findings and more. The project is a collaborative effort between [workplace 1 and workplace 2] and [researcher names], from [workplace 2], will be running the focus groups.<br><br>Poor maternal health status and behaviours have been demonstrated to have a negative outcome on offspring health. Therefore, the preconception period has been identified as an optimal time for intervention and women may also be more receptive to altering health behaviours at this time. It is hoped this will be of benefit to all [workplace 1] staff and students, regardless of life stage or gender. The Portal will also need to engage with women and men who are not looking to conceive, not just because an estimated 50% or pregnancies are unplanned but to encourage and empower all staff and students to take control of their health.<br><br>Input from all disciplines and backgrounds is very welcome. There are currently limited spaces for the lunchtime and afternoon sessions and several spots available for the morning session.<br><br>**Please R.S.V.P.** with your preferred session time by Monday 3rd September to [researcher name] at [researcher email] to participate.

Table S2. Focus Group Question Schedule

Introductory Question	What does preconception mean to you? At what point is a woman preconception?
Preconception and Pregnancy Health	What is important to you when thinking about preconception and pregnancy health; that is, what aspects of health should we focus on both prior to becoming pregnant and when pregnant?
	Do you/Did you have objectives to help your preconception or pregnancy health? What health objectives do you/did you have?
	What lifestyle health objectives do you know of that are there to assist women to conceive? Do you find these objectives easy (for yourself or others) to achieve or demanding (for yourself or others) to achieve?
Health in the Workplace	What aspects of work go counter to you achieving your health objectives? What kinds of things do you think your workplace could do to make it easier for you to meet your own objectives/public health objectives?
Healthy Lifestyle Behaviors	Have you been involved in any initiatives focusing on people meeting health objectives (diet programs, fitness programs etc.)?
	What motivated you to become involved in these initiatives? Are you still involved? If so, what motivated you to keep going? If not, what caused your motivation to wane?
Barriers and Enablers to Participation	What kinds of things allowed you to capitalize on those programs?
	What kinds of things stopped you from capitalizing on those programs?
Workplace Health Portal	Our aim is to develop a workplace portal to provide health information to women during the preconception, pregnancy, and postpartum periods. What kind of information or content do you think a portal like that should include?

Table S3. Codebook Informed by the COM-B Model

Hierarchy	Code	Description	Example Quote (relevant text in ***bold***)
Capability→Physical<br><br>Physical capacity to engage in a particular activity	Health behaviors	Physical actions, e.g., walking or prolonged sitting, that an individual performs that may lead to health benefit or detriment.	*I'm back to full time and I'm* ***not doing any exercise*** *inside or outside because I feel like between full time work and the baby, I have time for nothing else.*
	Health or disability status	An individual's subjective or objective level of physical wellness, illness, or disability. This includes biological or physiological states, symptoms and impairments, e.g., BMI or diabetes.	*I think* ***it's all your health system probably****, I think that's even when I was a child because my mother had difficult pregnancies. She instilled into me and my sister that* ***it was really important even as children to be really healthy,*** *to be active****, to have a healthy heart,*** *to eat well.* ***Because she said when you're pregnant everything impacts on your insides.***
	Skill development and training	A process of identifying gaps in skills and/or developing physical skills or abilities. Includes specific training provided to staff to participate in a wellbeing activity, e.g., marathon training, or related to OH&S matters, e.g. induction or first aid training.	***Exercise Science students will offer*** *free* ***training for the Launceston Ten, and that's been done every year for about the last five years.***
	Infertility	Inability or difficulty in conceiving. Includes references to age-related infertility and IVF.	***I do also worry a lot about the conceiving of the baby, see I'm 33 so by the time I'll be 34, possibly having a baby by 35*** *and then I would like more babies. So, I think* ***by the time I'm finishing with my***

			children and I'm fortunate enough to I will probably be in my 40s. And that worries me a lot, especially when there's lots of statistics about what happens when you become 35 and you're pregnant, that's a big worry.
	Preconception, pregnancy or postpartum (PPP) Status	The extent to which PPP status impacts women's ability to engage in healthy lifestyle behaviors, workplace activities or other tasks.	*You wouldn't want to start out with something like cross fit or … out of nowhere. Or hot Yoga for example, things that don't … of just* ***being aware of what's inappropriate during pregnancy that you wouldn't be able to continue once you fell pregnant****.*
Capability→Psychological<br><br>The psychological capability to perform the necessary thought processes needed to perform an activity, e.g., understanding	Barriers or enablers related to PPP at work	Work-related factors that assist or hinder the experience of being a PPP woman in the workplace.	In response to HR query regarding maternity leave:<br><br>*Well, she answered my question, but that was the end of the conversation.* ***There was no other sort of information about support services or anything in regards to [maternity leave].***
	Barriers or enablers related to work	Work-related factors that assist or hinder employee health, wellbeing, or progression in the workplace. Unrelated to PPP.	*But we're* ***too busy*** *the next week as you were saying.* ***The workload, the demand****...*

	Knowledge building	Refers to the intentional creation of cognitive concepts, e.g., thoughts or understanding, as a result of dialogue, questioning or improvement of ideas, e.g., reading a journal article. May be positive or negative, e.g., being "bombarded" by too much information.	*So, for example, in our experience, we were* ***reading a lot of books*** *as kind of you said. And I think that when I got to the second trimester of the pregnancy,* ***I gave up and said no that's too much. Like I don't want to know any more and I gave up actually****.*
	Mental health status or behaviors	An individual's subjective or objective level of mental wellness or illness. This includes biological or physiological states, symptoms and impairments, e.g., stress. Also includes responses that link actions, e.g., meditation or taking a lunch break away from your desk, with mental health or wellbeing.	*And I guess one of the things that is really helpful is* ***resilience. The resilience of some women, in spite of lives that you just could not believe.*** *Just so terrible. And* ***they come through it and then there are other women who, who don't, who have mental health issues and they don't come through it****.*
	Psychological impact of PPP status	Psychological factors, e.g., knowledge, that affect an individual's capacity to prepare for or engage in behaviors related to PPP. Includes mental health status and behaviors during preconception, pregnancy, or parenting.	*In my case, in terms of the psychological preparation,* ***I did feel like "when is it a good time?****" For example,* ***we ended up getting pregnant after I finished my PhD because I thought you know, I need this done before…I can't deal with everything at the same time.*** *I'm sure there's a lot of people that did it while having kids. And I'm sure they can do it but I felt that I couldn't.*

	Psychological wellbeing supports	Workplace or external supports, programs or activities that aim to assist with individual psychological wellbeing, e.g., mindfulness sessions.	*We talked about mental health. I did use the* ***[counselling] sessions that are offered to staff and I think they are three a year or something like that.*** *It was very straightforward and easy and I'm pretty grateful that I could access it.*
Motivation→Automatic<br><br>Automatic processes that give rise to and direct behavior including emotions, and impulses derived from associative learning or base personality	Behavior modelling	A method of behavioral influence, whereby an individual observes the behavior of another person and then imitates the behavior. Also refers to the comparison of behaviors, achievements, and actions between individuals.	*But at that time I was more aware of being healthier, so* ***I joined a group of people that would walk around the campus and would actually get out of the campus and come back****. And it turned out to be* ***one of those ladies was pregnant already, so we had a lot of conversations about being pregnant she was telling us things that she was doing. So that kind of was preparing me at the same time as I was taking the time to do exercise****.*
	Competition	Activities or situations where an individual or group is trying to win or compete with another individual or group. Could refer to competitive behavioral traits or social influence on competitiveness.	*We've had that, I mean we've had* ***walking challenges****, we had a couple of them a week here. Pretty much* ***everybody in my department took part at one stage with our little teams of four****, not only were* ***we competing then at then at a university level but also within the department****.*

	Habit formation	The process through which behaviors become automatic or 'habitual' through regular repetition.	***The first thing I do when I get to work is open my window****, so many people I know in my wing have closed windows…*
Motivation→Reflective<br><br>Reflective processes that give rise to and direct behavior such as planning, evaluating or goal setting	Beliefs	An idea, concept, or opinion that an individual accepts to be true. Often expressed in a factual manner without introducing elements of chance or doubt, e.g., modal verbs such as 'may', 'would' or 'could'.	***You can't trust in a fair process if you are a woman and you're liable to be pregnant and inconveniently out of the workplace****.*
	Changing attitudes or perceptions	Alterations to perceptions or beliefs over time, particularly with the introduction of new knowledge or experience.	*In those days that long ago,* ***I think even when probably when we were studying midwifery rather a few years ago. There might've been a little bit about preconception care, but it's a relatively new phenomenon.*** *There is so much information out there and so many things, you know, people have to be aware of lysteriosis and this and that and the other*
	Goal setting	The process of identifying an objective and (optional) establishing measurable goals and timeframes in which to achieve it.	***I've been thinking about…I'm struggling with at the moment with my health side.*** *Like I have to find a time, but* ***I've been thinking about getting a stand-up desk****…*
	Feedback	Information provided in response to a product or (in)action. Can be constructive or	*Can I join your team? Serious. I got this last year (points to exercise watch) for that very reason to*

		destructive and includes haptic or sensory feedback, e.g., ‘buzzing’ from wearable technology.	*just get to 10,000 steps a day. Cause* ***I realized I was doing, I got it and was like about three to four thousand steps a day, if that. Yeah. And so now it’s, yeah, I get a little ‘beep beep’ and you’re like “yeah ok, I need to move”****.*
	Incentives	A reward, usually monetary or concessionary, that motivates people to complete a task or improve a behavior.	*I went on one that someone did in the Menzies did three years ago where you wore a fit bit for a month and they tracked your activity. That's all.* ***You got to keep the Fitbit****, but then I stopped wearing it.*
	Perspective	A point of view or individual judgement of a situation, place, or thing. Often based on an individual’s own experience and usually expressed with less certainty than ‘beliefs’. Also includes personal interest, i.e., individual enthusiasm or passion for a particular activity or topic.	*…****everything has pros and cons, whatever you’re doing.*** *I think there are offerings at the university, there is the time problem there which I mean even I sometimes …* ***depending on the time of the year if I have deadlines, I don’t have time to go to the gym. But I think it’s more a personal motivation, whether the university can do something to motivate people to exercise is a different thing, but I think the opportunities are there for people to get active if they want to****.*

	Directed behaviors based on PPP status or PPP intent	The influence of PPP status on directing behaviors. Also includes the stage after postpartum, i.e., motherhood/parenting.	***When I started with the preconception of the idea that we were looking for a baby, I started joining…that's why I started walking more. I started finding out things that were happening in campus that I would normally not do.***
	Preferences	A liking for one thing or person more than another. Relates to belief but contains two components, i.e., "I believe option one is better than option two".	*Instead,* ***I consciously go for a walk because I don't like standing.*** *Because I stand still when I lecture a bit, yeah so* ***I'd rather go for a walk****.*
	Self-efficacy	An individual's belief (or lack of) that they can succeed in specific situations, achieve their goals, overcome barriers, or accomplish tasks.	*It's like the food, you know,* ***you have a choice and it's up to you whether you choose a sausage roll or the fruit or just bring your own.*** *That's right you are all in charge.*
Opportunity→Physical<br><br>Physical factors external to the individual that provide opportunities to engage in a behavior	Access to PPP supports	The opportunity to identify and avail of work-related supports designed to assist with work-family balance for PPP women, e.g., keeping in touch days for women on maternity leave. Does not include flexible work arrangements.	*I'm just thinking of one time I have been out of Tasmania since having a baby.* ***I was at a conference and I had the baby with me, and they were very good about family friendly, friendly things****…*
	Access to health opportunities	The opportunity to identify health needs and to obtain the service or take part in the activity that fulfils those needs. Does not relate to	*I've got* ***7,000 [steps] today, and that's just walking around my normal day****.*

		healthcare or medicalized supports nor does it relate to supports provided through social supports, e.g., family. Includes how well opportunities are coordinated/organized, access to trustworthy information sources.	
	Access to healthcare	The opportunity to identify health needs requiring medicalized care and to obtain the support or service that fulfils those needs. Refers to care provided by HCPs in the health system. Includes health information provided by HCPs.	*So, I'm* ***going to my doctor*** *to do that when I'm in Melbourne on Monday.* ***She's a great doctor and I have access to that****.*
	Access to in-person workplace programs	An activity, program, facility, or event that is provided free of charge or subsidized by the university for the benefit of staff health.	***Counselling is available generally; you get three sessions a year****.*
	Access to online or technology supports	The opportunity to identify individual needs and obtain the support or service to fulfil those needs in an online format. This may include smartphone access; internet access; smartwatches; access to online information, apps, or programs etc.	*Dissemination of information is the key issue here and I thought that perhaps* ***we could have a Mylo site where we can access all the information. There could be a health information section for staff wherever like a unit and it kind of introduces all of the staff to where all of the information is.*** *Rather than having like a shared health drive and then all of these other little things.*

	Accessibility	How much of a burden it places on the individual or whether an individual requires specific skills or capacity to access or engage with healthy lifestyle activities or supports.	*It's been in books and things, but then you have to be literate, don't you? You have to be able to read and* ***have to be able to access the information.***
	Activity variety or choice	Provision (or lack) of a selection of different or diverse wellbeing activities or options to choose from.	*Well, it's terrific, I mean as a staff member you can salary sacrifice...* ***wide range of classes, touted for a range of abilities and fitness levels.*** *And having something on campus just makes it very easy to go.*
	Affordability	Specific mention of the cost of wellbeing activities or supports.	*...we run a walking group every week. That's* ***free for anyone*** *just to come along and do a walk.*
	Awareness	Knowledge or perception of available wellbeing activities or supports.	***I only found out through friends*** *that have babies and work here.* ***I've never noticed that there was a baby's room next to the cafeteria until someone pointed out and as I started using it a lot or what bathrooms have changing facilities. There's no one place to go to find those things****, it's like people telling and passing that information on.*
	Financial (in)stability or access to finances	The opportunity and/or privilege (or lack thereof) to be financially secure and be able to	*...when you look at the research that is out there,* ***socioeconomics does directly impact on your child***

		pay for access to health-related opportunities. Includes references to socio-economic background or financial insecurity brought on by family status, i.e., single person household versus couple household.	***essentially**. Like in development milestones and things like that so yeah, especially in the age of information where it's all out there for you to find,* ***if you don't have a good socio-economic background then yeah absolutely.***
	Food environment	The physical presence, availability or means of preparing food, relative to the individual, that may affect food choices.	*I think that's the biggest thing, there is **limited [food] choice**. When I think back to **19 years ago when I was on this campus, there was a lot more choice**. And **there was a lot more fresh produce available** like I would go to the cafeteria and buy fruit and fruit salad.*
	Location of activity or wellbeing opportunity	Where the activity takes place or the location where the support is available, e.g., Sandy Bay Campus or Newnham Campus.	*Well, I think **one of the nice things about this campus is that because it's quite spread out you have to walk a little bit**, so you can get a reasonable amount of incidental exercise every day. And even if you've only got 15 minutes **there's always a tree you can sit under and have your lunch when the weather's nice**...*
	Loss or lack of resources	Enablers of wellbeing or support that were previously available to the individual but are now no longer available.	*But that could happen if you go part time, I mean **I had my own office for a long time and they put somebody in, somebody else in**, I don't particularly*

			like that. But you know, these things happen and it's about the requirement for space when you've got limited amount of space.
	Non-work environment	Relates to the physical, non-work environment that the individual inhabits or interacts with, such as the neighborhood or community.	*Yeah. My friend had a baby, it was in France and again, much more relaxed. Also, much more relaxed about the fact because it's* ***basically impossible in France not turning around with people smoking as well****. When I went to visit recently, we were like, yeah, like* ***outside at her place where people are drinking and everyone was smoking and they're like, oh, you can't sit here, and she's like, I live in France, like if I don't go places where people are smoking I can't go anywhere****.*
	Systems or policy enabling	A policy, e.g., staff agreement, or system, i.e., multi-level inputs or processes, such as the influence of policy on organizational decisions that in turn affect individual employees, that impact individual healthy lifestyle behaviors or wellbeing. Includes structural barriers or enablers.	*So, for me it would've seemed like* ***a logical step is once I contact HR*** *but then* ***they put me in the right direction or put me in touch with services that are available.***

	Time barriers	Specific mentions of time pressures, lack of time, being busy or workload. Also includes mentions of time needed to participate in a program or activity.	*Because* ***we don't have time****. We don't always sit there and think what do I really want to know? Because* ***you're just getting bombarded by so many things****.*
	Union	An external organization that helps protect employee rights and entitlements. Includes specific mentions of 'union'.	*There is some information about EBA and you can always,* ***if you're a union member, you can contact the union****. I think that's something that a lot of people do in this workplace, if they're unsure where to go.* ***They just contact the union and the union will often give them advice as to who to contact, which I think is nice that we do have that support.***
	University as community space	University engagement with the wider community through events, shared facilities, or knowledge dissemination.	*…good to have some other* ***options that will all be open to the public as well potentially because it's going to be much more of a community-focused campus****.*<br>*So, when you talk about the population not being high enough, that could be potentially not be as much of an issue because there will be* ***more people coming through the whole area to go to the museum or like even to go to the stadium for football games and things like that****. So, the cafes*

			can open on weekends, it will make it worthwhile for them.
	Work environment	Relates to the physical workplace environment (rather than the 'perceived' environment which may be conflated with workplace culture), predominantly OH&S issues. Includes access to facilities e.g., lunchroom.	*Then it's making sure that your area has a* ***big enough fridge for everyone to bring their lunch. These little bar fridges everyone brings their lunch and they're packed in there and it's not really accommodated in some areas for people to all bring their lunch and have a fridge.***
	Work flexibility	Providing (or not) flexibility to individuals with regard to scheduling their hours of work, thus allowing them to adapt their workday to fit with other needs or responsibilities. May or may not relate to PPP.	*It is really important to have that* ***flexibility for peace of mind that if you need to make an appointment or gym or just want to go have a lunch break or you want to leave early because you've got to go to someone's dinner or something that you can. If the boss says no 8:45 to 5:06 you cannot leave and your lunch break. You have to be back at that exact time****.*
	Workplace role	The effect an individual's occupational role may have on their physical opportunities for health and wellbeing. For example, a casual worker or a worker manning a reception desk may not be able to participate in the on-campus wellbeing activities. Also captures data	*And simultaneously it's really important to look after mental health.* ***They're not logically thinking it through what do all these messages, what messages that they're sending to everybody who is working here****, including people who are vulnerable in preconception or whatever. And* ***not***

		relating to job insecurity, e.g., casual work or fixed-term contracts.	***sure whether they're going to have a job, or you know.***
Opportunity→Social<br><br>The external social and cultural opportunities that lead to individual relationships and understanding of the world	Back to Work Transition	Returning to work after a pregnancy (or pregnancy loss, i.e., stillbirth) and the supports that are put in place to ease the transition. Also includes supports provided during a woman's maternity leave period.	*I would potentially like to have more time off, change my fraction and all those bits and pieces, but* ***I had a look at some of my friends that have their own businesses, and they have different jobs and things like that and I wonder if I want to continue breastfeeding past 12 months, how on earth am I going to achieve that?***
	Caring responsibilities	Duties or activities, including mobility or household tasks, undertaken by an individual to care for family members with specific needs, e.g., young children or family members with an infirmity.	*I had huge problems and then* ***my daughter was born she had huge health problems which went on for years*** *and I ran out of time to do my PhD.*
	Colleague attitude	The effect of colleague opinions, guidance and/or influence on individual behavior.	*That's a good question. I don't know who I would have gone to at the first place to get advice. I would probably, I had a good relationship with the head of school, which is not there anymore. But* ***if I wouldn't have had that good relationship, maybe I would have gone to a colleague that I have good relationship and say, what do I do?*** *Basically, I*

			think that that's what I would have done. So, I would definitely seek for that individual approach.
	Consolidated Approach	Lack of a unified, university-wide approach to employee health and wellbeing.	***[The workplace] does a lot of ad-hockery,*** *so you have things that [are] offered and it's someone who's got passionate and made it happen. And* ***you don't know if it's going to carry on, it's just...you don't know if it's consistent.***
	Family support	Guidance or physical/emotional support from family members, including partners, spouses etc. Includes learnings or experiences derived from family interaction.	*...in terms of the information, I'm just seeing my doctor and then* ***talking to my friends and my mother*** *and I think that's all the research I need. I might get a bit fanatical.*
	Health care professional (HCP) attitude	The effect of HCP opinions, guidance and/or influence on individual behavior.	*Like these days you can't bring it back to basics, but* ***I just think women are overloaded with the amount of information from doctors and their nurses****.*
	Management attitude	The effect of management or HR opinions, guidance and/or influence on individual behavior.	*But* ***in some way, I did feel protected by my head of school****. The fact that, you know, I went and said,*

			and ***he said, oh, it's great that you tell me, but let's keep [the pregnancy] quiet****.*
	Media	Information from traditional media, i.e., television and radio, and social media, e.g., Twitter and Facebook.	*I think there's a lot of bad images about pregnancy and birth out there. Because my oldest is now 18 and she just says to me, I'm never having children. And it's because* ***I think of what she gets influenced by on social media and Netflix and whatever else she's looking at.*** *And I think that maybe impacts on her because she loves kids, but she just says, I'm not going through all that. I really think* ***it's all those negative messages really impacting on that generation at the moment.*** *And again, that's a real worry.*
	Peer groups	Guidance or physical/emotional support from friends or colleagues. Includes learnings, influences, or experiences derived from friend/colleague interaction.	*But you had just made me think about something as well about the types of information that different people are able to provide to women.* ***When women talk to other women, they're looking for different information about perhaps thinking about having a baby*** *than they would if they were talking to their GP or a health nurse or a midwife as well. So, there are different levels of information for preconception as well.*

	Role of partners	The role, influence or support a partner adopts/provides to an individual during PPP or parenting.	*But I thought that, well* ***I need to know if I am actually seriously thinking about having a baby, considering my partner is studying full time and I'm the breadwinner.*** *I'm like, it's really important to know where I stand in regard to that.*
	Societal or gender expectations	The assumption or expectation that an individual conforms their behavior, appearance, or views to meet societal or gender norms.	*Which is very challenging with* ***this idea of expectations, perfection, what were you looking for? What is, you know, the 'yummy mummy' - perfect? You've got to get your beautiful body back, you've got to do it all really and have your great career, get pregnant when you want. Huge pressure.***
	Workplace champions	A formal (e.g., assigned role) or informal (e.g., voluntary, or initiative-led) role undertaken by individuals who are dedicated to supporting and driving a program or intervention and overcoming resistance to the resulting changes within the workplace.	*And it's reminding people like, we all know that a walking meeting is better than a sedentary meeting, but we forget. And think, oh, she was really good to just on that, walking around the campus. It was an easy conversation, but you forget, or you get too comfortable. So, I think it's reminding people all the time and having a central place for people like with a big notice board with bits and pieces on it. And people did use it. I know it's fallen by the wayside a bit, but people did use it.*

			So, ***I think having someone, I know it's a big responsibility for someone in each area, but if someone can and is passionate about it, like us. We can encourage people it more, to do things.***
	Workplace culture	Relates to the 'character' or atmosphere within the workplace. A summation of the values, traditions, beliefs, interactions, behaviors, and attitudes unique to a workplace. May be positive or negative.	*My friend had a similar experience, but it was a lot more positive. She works at the state library in Victoria and* ***she was asked to apply for a promotion. And at that point she was only six weeks pregnant and so she went to her manager and explained the situation. And said, look, I want to apply but I am six weeks pregnant. And she was completely supportive because she's like, you're applying, you're our top candidate. And she got the position straight away and announced literally in the next couple of days that she was pregnant to her colleagues and they had a big party.*** *So, it was much nicer.*
Other	Definitions of preconception	How individuals define or describe the preconception period or reproductive years.	*I would say that* ***it's a lifespan thing because reproductive health is influenced by what happens really well even when they're in utero themselves. So, I think it would be a fetal life-long thing.***

	Digital health resource for PPP women	Any direct references to a specific health in preconception, pregnancy, or postpartum digital resource, or online supports, or services that may integrate well into a specific digital resource.	*That sounds [like] you and I can* ***go back to the internet people and say here is some feedback from a strong woman advocate women's group. We don't want all [this] news, we've already said to you, we don't want [it]. We want something that's really gonna benefit us as a staff portal.***
	Preparations for pregnancy	An individual getting ready to become pregnant by making changes to their health behaviors, home life, work life, knowledge or having discussions with partners, friends, or family. Also includes the preparations of the partner.	*Lots of things. I'm thinking on a selfish level there are so many things I want to do,* ***I want to do my PhD*** *and I'm like, how is this actually gonna work?* ***Health of course, I want to get to a certain level of fitness.*** *So, then the yummy mummies, you know, like stupid concept of* ***being fit again, then all the preparation in terms of the vitamins.*** *But then* ***also my partner's health, so he's been trying to quit smoking for the last three years.*** *And I think that's a really important thing to do if we do fall pregnant beforehand.*
	Student Related	Responses relating to student wellbeing rather than employee wellbeing.	*I was just thinking what's really good on campus at the moment* ***some…Students have just started up the…Food Revolution Society. And they've got the community garden of the ground again and they want to start a veggie box scheme next year***

			and want to do some cooking classes with students who are our future*. So, we're starting to see some changes but of course they will all graduate at the end of next year, so hopefully the cycle will follow, but that's exciting. So, we are* ***starting to see some change happening within our student population too.***
	Weight Gain (or loss)	Discussions or experiences of weight gain or weight loss. Can refer to weight gain or loss in partners or other individuals.	*Often you hear about both partners in a couple going along that journey together. And that like* ***a certain portion of weight loss on the male so they can assist as well.*** *It's good for them to be in it together.*
